# Dysregulated Mucosal Immunity and Associated Pathogeneses in Preterm Neonates

**DOI:** 10.3389/fimmu.2020.00899

**Published:** 2020-05-15

**Authors:** Maame Efua S. Sampah, David J. Hackam

**Affiliations:** Division of Pediatric Surgery, Department of Surgery, Johns Hopkins University School of Medicine, Baltimore, MD, United States

**Keywords:** necrotizing enterocolitis, toll like receptors, sepsis, intestinal epithelial barrier, lymphocytes, regulatory lymphocytes, neonatal immunity

## Abstract

Many functions of the immune system are impaired in neonates, allowing vulnerability to serious bacterial, viral and fungal infections which would otherwise not be pathogenic to mature individuals. This vulnerability is exacerbated in compromised newborns such as premature neonates and those who have undergone surgery or who require care in an intensive care unit. Higher susceptibility of preterm neonates to infections is associated with delayed immune system maturation, with deficiencies present in both the innate and adaptive immune components. Here, we review recent insights into early life immunity, and highlight features associated with compromised newborns, given the challenges of studying neonatal immunity in compromised neonates due to the transient nature of this period of life, and logistical and ethical obstacles posed by undertaking studies newborns and infants. Finally, we highlight how the unique immunological characteristics of the premature host play key roles in the pathogenesis of diseases that are unique to this population, including necrotizing enterocolitis and the associated sequalae of lung and brain injury.

## Introduction

Early maturation of the immune system is a complex process that involves molecular, cellular and epigenetic programs. While *in utero*, the fetal immune system has traditionally been thought to exist in a sterile environment with no antigenic exposure (1) with a need for modulation to allow coexistence with the mother's immune system. However, a growing body of evidence suggests that the intrauterine environment may not be entirely sterile, as previously thought, and that the formation of a neonatal microbiome may originate *in utero* (2–4). Bacterial DNA has been found in the human placenta as well as amniotic fluid (5, 6), suggesting a unique placental microbiome that might impact the immunity of the fetus. While this area is still under active study, there is no question that the neonate becomes quickly exposed to a storm of pathogens immediately following birth. Importantly, the infant is inoculated with varying species of commensal microbiota as he or she passes through the birth canal. These initially include facultative aerobes such as *Escherichia* and *Enterococcus*, and subsequently obligate anaerobes, including Firmicutes such as *Clostridia*, Bacteroidetes, and especially *Bifidobacteria* (7). Evolution and variations in this commensal population play a critical role in shaping immunity and allergy, food digestion as well as brain and other bodily functions. Thus, the immune system must be appropriately primed to fight potential infections, while also modulating itself to allow for beneficial microbial colonization and to avoid potentially harmful inflammation and autoimmunity.

Initially, the innate immune system is mainly responsible for surveillance in the neonate, involving cellular players which include phagocytes, natural killer (NK) cells, antigen-presenting cells (APCs), humoral mediators of inflammation, and complement. This surveillance occurs while the components of the acquired immune system mature and gain antigenic experience. The importance of breastfeeding is evident, as breastfed infants are able to receive antibodies and antimicrobial components in breast milk that help prevent certain acute infections (8, 9).

While the relevance of environmental factors such as pathogens, commensals, and the maternal-fetal interface to development of the early immune system is clear, it is important to note that regulation of the immune response to microbial and environmental cues takes place at the genetic level. A large number of transcription factors control critical aspects of immunity such as hematopoietic cell differentiation, determination of myeloid and lymphoid cell fates, immune cell activation, expression of antimicrobial proteins and cytokines, expression of cell surface receptors, and the establishment of memory, to name a few. These transcriptional networks are well-characterized and involve factors such as GATA3, Tbet, Bcl6, NFκB, STATs, IRFs, and AP-1. Overall, a multifactorial mechanism prevails where both genes and environmental factors interact in shaping the immune system. Furthermore, it is now well-understood that post-transcriptional mechanisms regulating transcription factor activity, nuclear architecture, and epigenetic mechanisms are crucial in the development and differentiation of immune system and related pathologies. These mechanisms include DNA and histone protein methylation, acetylation and other modifications, nucleosome remodeling, as well as the formation of higher-order chromatin structures (10). The consequences of these transcriptional, post-transcriptional and epigenetic programs can be short-term or have lifelong implications.

Given the above, this review aims to examine immune system dysfunction in compromised newborns and the related increased risk of complications such as necrotizing enterocolitis. Data from studies investigating components of both the innate and adaptive immune systems will be presented, as well as the effect of the immature immune system on the risk of infections such as necrotizing enterocolitis.

## Innate Immunity

**Figure 1:**
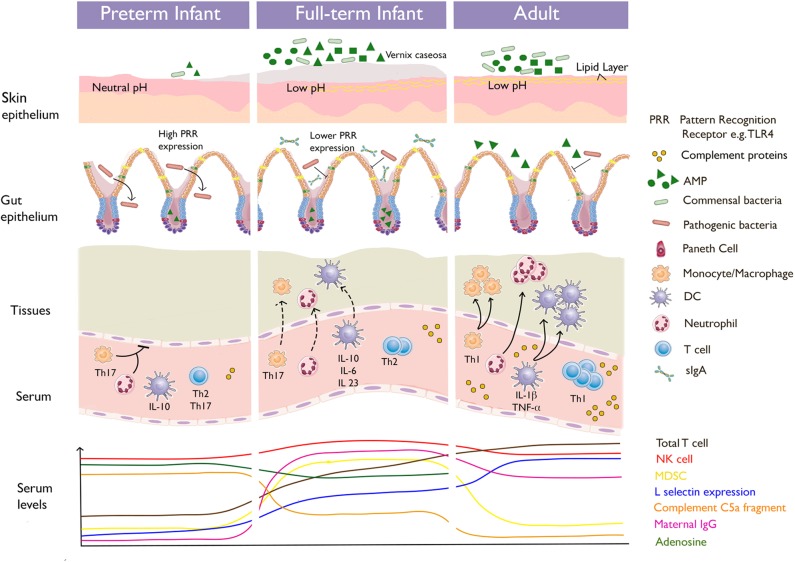
Diagrammatic overview of immune factors at their anatomic sites, illustrating how they interplay.

Innate protective mechanisms against pathogens are provided by the skin, respiratory and gastrointestinal epithelia, and other mucous membranes. These mechanisms are complemented by humoral factors, such as cytokines and complement components present in tissue fluids, blood, and secretions such as tears and saliva. These factors are present at birth and do not require gene rearrangements. The functions of innate immunity need to be both rapid (to prevent spread of the infection) and broad (enabling protection against multiple diverse pathogens at the same time). Soluble (e.g., complement and acute phase proteins) as well as cellular components contribute to this first level of defense. Important but often underappreciated determinants of immunity fall under this broad category, including immunosuppressive erythroid precursors, granulocyte/neutrophil function, and pattern recognition receptor (PRR)-based responses (see Figure 1).

### Physical Epithelial Barriers, Associated Signaling, and the Microbiome

Neonatal skin is easily disrupted and lacks the advantage of a protective lipid layer and acidic pH until ~1 month of postnatal age. This phenomenon is exacerbated in preterm infants, in whom it takes longer for these features to develop (11). The vernix caseosa, a naturally occurring biofilm that covers fetal skin, functions as a barrier against water loss, regulating temperature, and preventing microbial access. Development of the vernix caseosa begins in the third trimester, hence, it is often not fully developed in premature infants. It has also been shown that neonatal skin keratinocytes, and particularly the vernix, constitutively produce a broader array of antimicrobial peptides (AMPs) compared to older infants and adults (12) which provides an extra level of protection. AMPs generally include α-defensins and β-defensins and the cathelicidin LL-37, which have direct antimicrobial activity against gram-positive and gram-negative bacteria and some fungi, as well as the influenza virus, respiratory syncytial virus (RSV); and protozoa. These defensins and cathelicidins destroy pathogens by insertion into the membranes of a broad range of gram-positive and gram-negative bacteria, fungi, protozoa, spirochetes, and enveloped viruses (1). Once inside the microbial cell membrane, they form pores allowing the passage of anions through the membrane, thus depolarizing and killing the organism (13). The immaturity of premature skin is exacerbated by the iatrogenic insults inflicted as a part of lifesaving intensive care.

The neonatal skin epithelium is also rapidly colonized by a normal flora of commensal bacteria following birth that help to prevent colonization by pathogens (14, 15). Coagulase-negative staphylococci such as *Staphylococcus epidermidis*, micrococci, and other species constitute the majority of this flora, and have been shown play a protective role in the skin by secreting lipopeptides that bind to toll-like receptor 2 (TLR2) on neonatal keratinocytes and stimulating them to produce the AMPs hBD-2 and hBD-3. These features are not functional in preterm infants (16).

Like the skin epithelium, the epithelial surface of the neonatal stomach also lacks an acidic pH, which is thought to facilitate the establishment of commensal flora (14) mainly belonging to the phyla *Firmicutes* and *Proteobacteria* (17). AMP-producing Paneth cells are decreased in number in the small intestine of preterm and, to a lesser degree, term neonates, which may increase the risk of enterocolitis and invasion by pathogens. Some animal models have demonstrated more robust production of antimicrobial peptides by intestinal epithelial cells which may counteract this phenomenon, but this has been yet to be confirmed in humans *in vivo* (18).

In a full-term infant, enterocytes within the gut epithelium sample and identify antigens introduced into the intestinal lumen, signaling to intraepithelial lymphocytes via PRR (19–21) such as toll-like receptors (TLRs) and nucleotide-binding oligomerization domains (NODs). These receptors recognize antigens on pathogenic bacteria and elicit an immune response against infection.

It has been shown that higher levels of innate immune receptor expression in premature neonates compared to full-term controls lead to increased inflammation within the gut epithelium, leading to loss of epithelial integrity, and subsequent introduction of pathogens into circulation (22–24). This is often in the setting of an increased number of activating mutations in the signaling pathways associated with these receptors. For instance, TLR4 hyperactivation in premature mice and humans has been shown to lead to increased enterocyte apoptosis, reduced enterocyte proliferation and migration, and the eventual breakdown of the intestinal epithelium (25–28) that is a hallmark of necrotizing enterocolitis (NEC). Further, we have shown that TLR4 activation can reduce expression of endothelial nitric oxide synthase (eNOS) in the intestinal endothelium, causing decreased blood flow and ischemia that exacerbates the clinical course of NEC (29).

The gut epithelium, similar to the skin, houses cells that also produce defensins and cathelicidins. Intestinal epithelial cells (IECs) secrete β-defensins (hBD1, 2, and 3) (30–32) while Paneth cells secrete lysozyme, phospholipase A2, the AMPs, defensins (α and β), and cathelicidins (33, 34) in response to microbial or cholinergic stimuli. This creates a relative sterile and protected intestinal crypt environment. Microscopic and molecular analysis of tissue from non-viable fetuses and adults has demonstrated that Paneth cells are normally present by 12 weeks gestation, antimicrobial defensins at 13 weeks and lysozyme at 20 weeks (35–37). However, premature infants have been shown to have few Paneth cells with decreased antimicrobial producing function (36, 38).

Another parallel between skin and gut epithelium is the presence of commensal bacterial flora. The immune system is able to distinguish these microbes from harmful pathogens in part by limiting the location of innate immune receptors. For instance, in the full term gut, intestinal epithelial cells normally express few or no TLRs on their luminal surface (22), where they are in contact with commensals. Pathogenic microbes that invade through the epithelial cell layer are however recognized by endosomal TLRs, cytosolic innate immune recognition receptors, and TLRs located on the basolateral surface of epithelial cells, triggering an inflammatory response. On the other hand, commensal bacteria are able to inhibit signaling and inflammatory mediator production downstream of these receptors or induce anti-inflammatory cytokine production, thereby actively suppressing gut inflammation. However, the underdeveloped preterm intestinal epithelium is highly permeable and more easily colonized by pathogenic bacteria because of reduced gastrointestinal motility as well as limited enteric nervous system function (39), all of which set the stage for destructive dysbiosis, chronic inflammation, and microbial translocation through the weakened intestinal barrier, leading to potentially lethal diseases of prematurity. Additionally, the premature intestinal epithelium expresses high levels of TLR4, which causes an overreaction by the host immune system to gut bacteria that leads to excessive inflammation (25, 40). The elevated TLR4 expression in the premature gut is explained by the non-immune role that we discovered for TLR4 in the regulation of gut development, through its activation of Notch and Wnt pathways (41–43). Thus, in the relatively sterile environment of the fetus, TLR4 serves a predominantly developmental role, while the premature infant, in which TLR4 expression remains persistently elevated, mounts an exaggerated inflammatory response to bacteria upon colonization of the intestine by microbes (44). This elevated inflammatory state leads to mucosal barrier breakdown, bacterial translocation and the development of NEC in the premature host (45). Based upon these findings, we have embarked upon a strategy of TLR4 inhibition for the prevention and treatment of NEC, and have discovered a novel class of TLR4 inhibitors to serve as potential therapies (46, 47). We also note that breast milk, which is a powerful material capable of reducing NEC, is rich in molecules that inhibit TLR4 signaling, explaining in part their mechanisms of action in achieving NEC protection (48).

The respiratory epithelium is also known to express TLRs and the AMPs, SP-A, and SP-D, which mature in the last trimester of fetal development (49). Preterm infants therefore may lack these defenses, which is exacerbated by reduced numbers of resident alveolar macrophages compared to term infants. NEC-induced lung injury is particularly severe as compared to the lung injury that develops in premature infants who do not develop NEC. We have shown that TLR4 expression on the lung epithelium is required for the recruitment of proinflammatory neutrophils into the lung through the upregulation of CCL25 (50, 51), and that strategies to either inhibit TLR4 via the administration of aerosolized inhibitors, or through genetic deletion, can serve as novel lung protective strategies in the setting of NEC (50).

### Extracellular Components

In response to infection and inflammation, multiple mediators in the plasma are activated to fight pathogens. These mediators include the complement and kinin systems, mannose-binding lectin (MBL), fibronectin, coagulation factors, arachidonic acid metabolites, amines, and lysosomal enzymes. Many of these mechanisms are known to be impaired in the neonate, and more-so in compromised newborns.

#### Complement

The complement system, composed of three pathways for pathogen recognition, subsequent permeabilization, opsonization, and lysis of harmful microbes, also plays a significant role in priming the adaptive immune system. These include the classic, alternative, and the lectin pathways. Complement expression may vary in newborns secondary to common genetic variants as well as rare deficiencies. Fetal complement synthesis is detected as early as 6 weeks gestation, with gradual age-dependent maturation (52). Levels increase after birth and reach adult levels between 6 and 18 months of age (53). Serum complement activity is known to be decreased in term newborns compared with adults and further diminished in preterm infants. Levels of complement proteins in pre-terms, specifically C3 and C9 have been measured to be as low as 10% of adult levels, remaining low until up to 1 year of age (54). These components are known to be responsible for recognition of polysaccharide antigens and formation of the membrane attack complex in bacterial lysis, respectively. On the other hand, the complement activation product, C5a, a strong chemoattractant peptide and a mediator of mesenteric ischemia/reperfusion injury is found to be highly expressed in cases of NEC and is under study for its utility as a clinical marker for diagnosis of infants with NEC in combination with radiographical findings (55).

#### The Lectin Pathway

The lectin pathway of complement recognizes conserved carbohydrate moieties on pathogens, leading to opsonization and phagocytosis in an antibody independent manner, and making it an important pathway in neonates, who are relatively antibody deficient. This pathway is mainly activated by mannose-binding lectin (MBL) which serves as an opsonin for the ingestion of gram-negative and gram-positive bacteria by neutrophils and monocytes. Baseline polymorphisms are known to exist at the MBL locus, leading to reduced circulating MBL levels in approximately one-third of the population. However, levels of serum MBL have been measured to be about 70% that of adults in term infants and 5% that of adults in premature infants (56). Low MBL levels have been associated with pneumonia and sepsis in premature infants. Mannose-binding lectin (MBL) recognizes microorganisms and activates the complement system via MBL-associated serine protease-2 (MASP-2), which in a small case control study was found to be in higher concentrations in cord blood levels in premature infants predisposed to NEC and associated with a 3-fold increased risk to develop NEC (57). Given that extremely low MASP-2 concentrations was found in most premature neonates overall in the study, authors concluded that MASP-2 deficiency may represent a protective mechanism against excessive proinflammatory stimuli during the neonatal period.

#### Acute Phase Proteins

Acute phase proteins (APPs) are released by the liver, leukocytes, epithelial cells, and mucosal sites (58) in response to infection and trauma to resolve inflammation. Some of these APPs exhibit antimicrobial activities similar to antimicrobial peptides (AMP). APPs bind to pathogens and permeabilize their membranes. They are also capable of binding and neutralizing microbial toxins (59). Maturation of soluble APP is age-dependent, and preterm newborns have been found to be deficient (60), as is the case with levels of fibronectin, a glycoprotein that promotes neutrophil adherence to endothelium as part of their migration from blood to the tissues. This deficit likely results in reduced neutrophil function and increased susceptibility to bacterial infections, which is discussed further in the following section.

### Cellular Components

The workhorses of the innate immune system are comprised of the cellular components that include granulocytes (particularly neutrophils), monocytes, macrophages, dendritic cells (DCs), and natural killer (NK) cells. The cells generally phagocytose microbes, present antigens, and are responsible for killing pathogenic organisms.

#### Monocytes and Macrophages

These innate immune system antigen-presenting cells that secrete inflammatory mediators, perform their function by phagocytosis of microbes and subsequent antigen presentation to T and B cells, linking the innate and adaptive arms of the immune system. Following release from the bone marrow, monocytes circulate in the bloodstream and then differentiate into macrophages as they enter tissues. They subsequently become resident throughout the body, becoming specialized as distinct populations in the alveoli, interstitial connective tissue, bone, brain, and liver (58). There, they play the important roles of phagocytosis, killing microbes, producing cytokines and AMPs, clearing dead host cells, and antigen presentation. Neonates have comparable numbers of monocytes to adults (61). However, preterm monocytes have been found to be defective in their ability to be recruited to sites of inflammation via chemotaxis (62).

*In vitro* analysis of cells derived from preterm neonates have also demonstrated impairment in phagocytosis, as well as low expression of costimulatory molecules such as MHCII, CD40, and CD80 required for antigen presentation, a finding which has been associated with increased incidence of sepsis (63, 64). Other receptors such as TLR-4, CD14, and MD-2 which, together as a complex on the extracellular surface of macrophages, are involved in inflammatory signaling via LPS, an antigen derived from the wall of gram-negative bacteria. Neonatal cells appear to have normal levels of each of these molecules. However, the consequences of TLR activation in preterm infants and neonates are different compared to adults. Downstream cytokine response from interaction of LPS with these molecules in adults is consistent with a proinflammatory Th1 profile leading to expression of interferon gamma (IFNγ), IL-12 and tumor necrosis factor alpha (TNFα), which predominantly target intracellular pathogens. This response is different in neonates and especially preterm infants, where a Th17 dominant profile is observed, with IL-6 and IL2-3, which defend against extracellular bacterial and fungal pathogens, being produced. The anti-inflammatory and immunoregulatory cytokine IL-10 is also seen to play a dominant role. It is thought that this polarization prevents excessive production of the proinflammatory cytokines such as TNFα and IFNγ, which are associated with spontaneous abortion and intrauterine growth retardation (65, 66). However, this pattern of polarization nonetheless sensitizes preterm infants and newborns to infection by a broad range of intracellular micro-organisms which would normally require Th1 mediated clearance, such as *Listeria monocytogenes* and herpes simplex virus (HSV) (66). Interestingly, studies looking specifically at the neonatal response to the latter pathogen have demonstrated that an overly vigorous immune response via proinflammatory cytokines IL-6 and IL-8 may also occur, which is associated with an exacerbated clinical course (67, 68). In this regard, we have found that TLR4 signaling on the premature newborn epithelium leads to the differentiation of immature lymphocytes into Th17 cells, leading to the release of IL-17, and subsequent injury to the intestinal mucosa (28). Accordingly, strategies which inhibit IL-17 signaling were found to significantly attenuate NEC in pre-clinical models (28).

Levels of early response cytokines produced by innate cells such as macrophages are modulated by the neonatal metabolic state. The preterm infant metabolic state is characterized by stress induced by low oxygen tension that leads to increased expression of proinflammatory cytokines such as IL-6 and IL-8 via a HIF independent pathway (67). However, the response to low oxygen levels also involves a rise in adenosine levels. Adenosine is produced by cells in response to stress via breakdown of adenosine triphosphate (ATP) and is hence found to be elevated during hypoxia. This molecule has been well-studied and been found to play an immunomodulatory role through inhibition of TLR-mediated proinflammatory cytokines including TNFα, IL-12, and MIP1α (69). Specifically, in pre-term and term neonates, adenosine has been shown to downregulate proinflammatory/Th1 cytokine responses, instead mediating an alternative acute-phase response pathway via MBL, soluble CD14, C-reactive protein, LPS-binding protein, and the anti-inflammatory IL-10. Hence, in preterm neonates, adenosine attenuates pathologic inflammation by downregulating the inflammatory Th1 pathway. Recent studies have shown that administration of a probiotic *Lactobacillus reuteri* increases serum levels of adenosine and Tregs and results in lower susceptibility to NEC in stressed newborn mice by inhibiting the TLR4-mediated NFκB pathway (70–72).

#### Dendritic Cells

Like monocytes and macrophages, dendritic cells link the innate and adaptive immune responses (73–75) by serving as the main APCs for naïve T cells. DCs are classified into the plasmacytoid DC (pDC) and conventional DCs (cDC) groups. pDCs represent a small subset of DCs that circulate mainly in blood and lymphoid, producing massive amounts of type I IFN (IFNα/β) upon recognizing foreign antigens (74). They then acquire the ability to present these antigens to T cells (76). cDCs refer to all DCs other than pDCs. They mainly circulate in tissues, constantly acquiring antigens and have superior antigen processing and presentation functions. They produce IL-12 p70, which is strongly pro-inflammatory. DC-like cells are detected in the human fetal thymus, liver and lymph nodes as early as 12 weeks of gestation (77). In cord blood, the pDC: cDC ratio is 3:1 compared to a 1:3 pDC-cDC ratio in adults (78). Much like monocytes, DC populations in preterm infants and neonates are found to express lower MHC-II, CD80, and CD86 compared to adult cells, reflective of their defective ability to fully activate antigen specific T and B cell responses. As a consequence, neonates and especially preterm infants have impaired immune responses to most vaccines (74, 79, 80).

In parallel with the monocyte and macrophage populations described above, TLR expression in preterm, full-term and adult DCs have been found to be generally equivalent. However, in response to stimulation, preterm infant DCs induce production of the anti-inflammatory cytokine, IL-10 compared to term infant DCs that produce elevated levels of IL-10, IL-6, and Th17 inducing IL-23 (81). This cytokine production in term neonates declines over the first year of life while levels of pro-inflammatory cytokines such as IL-1β and TNFα increase. Further, neonatal pDCs exhibit severe defects in IFNα/β production upon TLR activation.

Neonatal lungs have been found to contain fewer cDCs, and a markedly lower number of pDCs in comparison with adult lungs (82). In laboratory studies, neonatal pDCs responded poorly to respiratory syncytial virus (RSV), a common pathogen encountered by neonates. pDCs from premature neonates have been found to mount a weaker response than those from full-term neonates to both RSV and to TLR9 agonists (83). The lower numbers of pDCs in preterm neonates likely translate into compromised antiviral function.

A specific population of cDCs that express CD103 are known to drive the induction of the chemokine receptor CCR9 and alpha4 beta7 integrin, both known as gut-homing receptors. CD103(+) DCs also contribute to control inflammatory responses and intestinal homeostasis by fostering the conversion of naive T cells into induced Foxp3(+) regulatory T cells. These cells have been found to be missing in neonatal gut tissue, resulting in susceptibility to *Cryptosporidium parvum* infections (84) and increased prevalence of food allergies (85).

#### Neutrophils

Neutrophils belong to a group of white blood cells known as granulocytes that have cytoplasmic granules containing cationic AMPs. They are present in the fetal liver parenchyma as early as week 5 of gestation (86). In response to stimulus, neutrophils must travel from the bloodstream to the site of inflammation, enter the tissue via diapedesis, phagocytose the pathogen, and kill it in its phagolysosome. Preterm neutrophils have been found to have deficiencies in each of these functions (87). First, selectin mediated rolling occurs at the vascular endothelium, which is required for neutrophil entry from the bloodstream into tissues. Compared with adults, neonatal neutrophils express <50% L-selectin on their cell surface compared to adult neutrophils. Preterm endothelium has decreased P-selectin expression compared to term infants (88). β2 integrin expression is required for arrest of rolling and adhesion to the endothelium but these are decreased on preterm neutrophils and are unable to be upregulated in response to stimulus (89). Diapedesis through the endothelial lining requires that the neutrophil actin cytoskeleton undertake significant structural reorganization, which neonatal neutrophils are unable to achieve (90). Levels of opsonins such as immunoglobulin G (IgG), complement, and their receptors required for antigen recognition and phagocytosis are reduced in preterm neutrophils (91, 92). This phenomenon of diminished opsonization in preterm neutrophils has been demonstrated in *in vitro* studies that show impaired adult neutrophilic phagocytosis following incubation in preterm serum (93).

Pathogenic killing in the neutrophil phagolysosome occurs primarily via an NADPH oxidase-dependent respiratory burst. Term neonates have been shown to have a largely intact respiratory burst, however preterm neonates, especially those that are critically ill (94), display decreased respiratory burst and killing on exposure to group B *Streptococcus, Staphylococcus*, and *Pseudomonas* (95). Other bactericidal molecules normally found in neutrophilic granules, such as lactoferrin, myeloperoxidase and BPI are also decreased in quantity in neonatal neutrophils (~30–50% of adult levels) (11), and more so in preterm infants, a phenomenon that has been correlated with increased risk of NEC (96, 97). This is also thought to confer susceptibility specifically to *Pseudomonas aeruginosa, Staphylococcus aureus*, and some strains of group B streptococci based on *in vitro* assays (98, 99). Neutrophils are able to form neutrophil extracellular traps (NET) by extruding DNA, chromatin and antibacterial proteins in order to sequester bacteria. NET formation has been found to be diminished by inhibitors present in cord blood of preterm and term neonates (100). Recent studies have examined whether NETs play a role in NEC pathogenesis. For instance, in one study, protein arginine deiminase (PAD) inhibited mice, which are incapable of producing NETs, were found to be protected from NEC compared to controls in a NEC model (101). Similarly, human NEC intestinal samples appeared to have increased neutrophil activation and NET formation.

Finally, neonates are unable to ramp up robust neutrophil production in response to infection, mainly due to a diminished bone marrow pool. This deficit is exacerbated in premature infants (102), in whom neutropenia is a clinical indicator of poor prognosis in cases of bacterial sepsis (103). Unfortunately, clinical trials for granulocyte colony-stimulating factor (G-CSF) or granulocyte-macrophage colony-stimulating factor (GM-CSF) as either prophylaxis or therapy for neonatal sepsis resulted in increased cell counts with no concurrent reduction in mortality (104).

#### Myeloid-Derived Suppressor Cells

Related to neutrophils and monocytes are a population known as myeloid-derived suppressor cells (MDSCs), which have been found to play a major regulatory role in inflammation and immune function in many pathological conditions (105–108). They are distinct from the former immune cell populations in their morphological, phenotypic, and functional heterogeneity. They produce high levels of ROS, NO, arginase (ARG1), an immunosuppressive enzyme as well as prostaglandin E2 (PGE2). They have also been found to highly express a number of anti-inflammatory cytokines, including IL-10, all of which mediate their potent inhibition of immune responses from T cells, B cells, and NK cells (109–111).

A recent *in vivo* study examining PBMC from preterm and term neonates determined that the levels of MDSCs in the blood of preterm infants was substantially lower than that observed in full-term infants (112). More so, low numbers of MDSCs in preterm infants was associated with the development of NEC. MDSC levels were also correlated with serum lactoferrin levels. Finally, *in vitro* treatment of newborn neutrophils and monocytes with lactoferrin converted these cells to MDSCs. These lactoferrin-induced MDSCs improved survival following treatment of newborn mice. Taken together, these findings suggest an important clinical and therapeutic role for MDSCs in disorders such as NEC.

#### Natural Killer Cells

NK cells play a significant role in defense to virus-infected and malignant cells by expressing receptors that mediate killing of these harmful cells. The percentage of NK cells in cord blood from preterm and term neonates is often slightly lower than in the blood of children and adults; however, the absolute number is slightly higher, due to overall higher lymphocyte count in infancy (113). Fetal and neonatal NK cells are mainly deficient in IFNγ and TNFα production and exhibit reduced cytotoxic function compared to adult cells.

Like cytolytic CD8^+^ T cells, NK cells mediate cytotoxicity, though they differ in accomplishing this via an MHC independent mechanism (114). CD56 is an NK cell-specific marker whose presence on the cell surface reflects cytolytic function. About half of neonatal NK cells do not express CD56, corresponding to a 50% capacity of full-term and premature infant cord blood NK cells to mediate cytolysis (measured at 15–60% in various studies) compared to adult NK cells (115–117).

NK cells kill infected target cells that are coated with IgG antibodies in a process known as antibody-dependent cellular cytotoxicity (ADCC) (117). Neonatal NK cell ADCC activity has been measured at ~50% that of adult NK cells. This phenotype is rescued upon addition of cytokines such as IL2, IL12, IL15, and IFNγ *in vitro* (118). Similarly, when exposed to HSV, IFNγ production is identical in neonatal and adult NK cells (119). These studies suggest the neonatal NK cell ADCC activity *in vivo* may be comparable to adult levels in the setting of an appropriate stimulus.

In general, there is scarcity of data examining the role of NK cells in NEC, however, one small prospective study found that preterm infants with NEC showed a reduction in their NK cell proportion compared to controls (120).

## Adaptive Immunity

The adaptive immune system consists of a cell-mediated response involving T helper cells (CD4^+^) and cytotoxic T cells (CTL, CD8^+^), humoral responses involving immunoglobulins and immunoregulatory actors including T regulatory cells (Tregs).

### T Cells

CD4^+^ T cells, known as “helper cells,” function by activating other lymphocytes to kill infected cells. After being presented with antigens by MHC class II molecules expressed by APCs, they produce cytokines that regulate the immune response. Depending on the kind of stimulus and resulting cytokine environment, they may differentiate into Th1, Th2, Th17, or Treg cells. Th1 cells mediate cellular immunity, Th2 cells are involved in humoral immunity, while Th17 cells produce the proinflammatory cytokine IL-17. Tregs are immune suppressor cells. CD8^+^ or cytotoxic T cells kill infected cells and cancer cells directly via antigen recognition using class I MHC molecules.

There are several features of preterm T cells which limit their function. First, preterm neonates have been found to have marked lymphopenia (up to 50% reduction) with a significant decrease in the percentage of total, CD4^+^, and CD8^+^ lymphocytes compared with full term infants (121). The reduction is most notable among the CD8^+^ population, resulting in an increased CD4/CD8 ratio.

DC and macrophages induce the production of IL-12 after encountering antigens. IL-12 in turn stimulates NK cells and induces naive CD4^+^ T cells to become Th1-type effector cells which produce IFNγ, initiating the expression of proinflammatory cytokines, such as IL-1β, TNFα, and further upregulation of IL-12 production (122, 123). Preterm naive CD4^+^ T cells have reduced activation and impaired early Th1 differentiation including IFNγ production (124). Upon encountering stimuli, these T cells express a Th2 and Th17 polarization, weak Th1 polarization, and low innate antiviral type 1 interferon responses (65, 125). They are therefore referred to as Th2 skewed (126). IFNγ production by stimulated naive cord blood CD4^+^ T cells has been measured as 5 to 10-fold less relative to adult CD4^+^ T cells, resulting in susceptibility to viral infections such as human cytomegalovirus (HCMV) and HIV (127). The transcription factors T-bet, GATA3, and RORγt, regulate differentiation into Th1, Th2, and Th17 phenotypes, respectively. Accordingly, recent studies have shown that the proportion of T-bet expressing CD4^+^ T cells is reduced within the preterm T cell population (121).

Given the relatively preserved Th2 response, preterm T cells are still able to provide help to newborn B cells for antibody synthesis. CD8 function is also relatively intact in the preterm infant, with IFNγ production by stimulated naive cord blood CD8^+^ T cells comparable to adults' (127). It is unclear if the marked reduction in CD8^+^ T cell frequency, contributes to the increased risk of infections in these extremely premature neonates.

Tregs, which suppress fetal anti-maternal immunity and persist at least until early adulthood are abundant in the peripheral blood and tissues of the human fetus and preterm infant. *In vitro* studies looking at cord blood have shown no quantitative differences within the Treg compartment between full term and preterm neonates. However, Tregs are also involved in T cell migration to tissues such as skin and gut in a process that depends on the expression of the homing receptors CCR9/α4β7 and CCR4, respectively. CCR9 signaling is also known to regulate the immune response by inhibiting Treg development (128). The preterm Treg cell compartment has been shown to have lower frequency of α4β7-expressing but higher proportions of CCR4- and CCR9-expressing cells compared with full-term infants. This reflects an altered homing capacity of T cells to their target tissues in preterm infants compared to full-term. The premature newborn intestinal mucosa is characterized by an abundance of proinflammatory IL-17-producing Th17 cells which comes at the expense of anti-inflammatory Foxp3^+^ Treg cells, and the relative skew toward a pro-inflammatory state contributes to the excessive inflammatory response that leads to development of neonatal necrotizing enterocolitis (28).

### B Cells and Immunoglobulins

The B cell receptor is made up of antibodies specific for antigen detection. Upon binding of the antigen to the receptor, the former is endocytosed, processed, and presented on the B cell surface by MHC-II proteins which bind to a helper T cell. This triggers T cell activation, cytokine release to induce B cell proliferation and differentiation into antibody-producing plasma cells or memory cells. Antibodies that encounter antigens neutralize the associated pathogens and/or attract macrophages or killer cells to attack them.

Passive transfer of antibodies to the fetus and newborn occurs via transfer of maternal IgG from the placenta or secretory IgA (IgA) from breast milk. *In utero*, fetal serum immunoglobulin concentrations are significantly low until 18–20 weeks of gestation. Concentration of fetal immunoglobulins rises with the transfer of maternal immunoglobulin G (IgG) across the placenta during the third trimester of pregnancy. Preterm infants at <22 weeks gestation have 10% the level of maternal antibodies, increasing to 50% by 28–32 weeks, and elevating to 20–30% above maternal levels by term (129). This lower level of IgG compared to term neonates is likely due to less time for transfer, lower production levels and impaired placental transport. Antibodies from these infants therefore demonstrate low opsonic activity for all types of organisms (130). IgG concentrations may drop further after birth in these preterm infants due to the normal physiologic hypogammaglobulinemia that occurs in all infants. However, breast milk from mothers of preterm infants have been found to have higher levels of sIgA compared to term mothers' milk (131–133). Clinical trials evaluating the effect of oral immunoglobulin administration in preterm infants (134) have found no effect of oral immunoglobulin administration on risk of immune mediated conditions such as NEC. This is of interest, given that a recent study using a mouse NEC model showed that secretory IgA from maternal milk was protective for NEC (135). This data was correlated with levels of secretory IgA levels from preterm infant fecal samples.

In spite of limitations in the quality and quantity of immunoglobulins, even premature infants as young as 24 weeks gestation respond vigorously to protein vaccines (136, 137) such as tetanus and diphtheria toxoids, hepatitis B surface antigen, and OPV (138, 139). In contrast, responses to polysaccharide, T cell-independent antigens, such as the capsular polysaccharides of *Haemophilus influenzae* type b or Group B streptococci, are severely blunted in both preterm and term neonates until ~18–24 months (140). Pneumococcal and *H. influenzae* conjugate vaccines were designed as a solution to this phenomenon of poor response to polysaccharide antigens. In complexing polysaccharide antigens to immunogenic proteins, a T cell mediated mechanism is required (141).

## Immature Immunity and Disease

As discussed above, the lack of maturation of intestinal innate and adaptive immune defense mechanisms in premature infants explains their susceptibility to diseases of infectious and inflammatory etiology such as NEC (see Table 1). As demonstrated, the components of adaptive immunity regulate the innate immune system which can cause disease when allowed to respond unchecked. Preterm infants, who are born with underdeveloped adaptive immunity also have reduced transfer of maternal antibodies, especially formula fed infants (142) placing them at greater risk for inflammatory diseases such as NEC. The role of dysfunctional TLR4 signaling and other immature immune activation, compromised barrier function as well as deficits in humoral and cellular immunity have been discussed elsewhere in this article.

**Table 1:** Summary of differences in development between preterm infant, term infant and adult immune components.

	**Preterm infant**	**Full term infant**	**Adult**
Skin epithelium	Thin epidermis	More developed	Normal
	No lipid layer	Lipid layer present	Normal
	Neutral pH	Acidic pH	Acidic pH
	Vernix caseosa develops late	Vernix caseosa present	Not present
	–	Broad array of AMPs	Less AMP diversity
	Keratinocytes underdeveloped	Commensal bacteria interact with keratinocytes to make AMPs	–
Gut epithelium	Higher levels of PRR expressed on epithelial cells	Fewer PRR	Normal levels
	Few Paneth cells; decreased AMPs	Paneth cells make lysozyme and AMPs	Paneth cells make lysozyme and AMPs
	Epithelium more permeable to pathogenic bacteria	Epithelium more resistant to pathogenic bacteria	Normal
Complement	Low levels	Increased levels	High levels
	High level C5a fragment	Lower levels	Low levels
MBL	Low 5%	10%	100%
APPs	Low soluble APP	Increased	High
Monocytes	Comparable levels	Comparable levels	Comparable levels
	Cannot be recruited to tissue	Recruited to tissues, but fewer tissue macrophages than adult	Normal
	Poor phagocytic ability	Normal phagocytic ability	Normal
	Low receptor levels	Normal receptor levels	Normal levels
	Trigger Th17 response	Trigger Th17 response	Th1 response
Metabolic state	Low O_2_ tension	Low O_2_ tension	Normal O_2_ tension
	Proinflammatory cytokines	Proinflammatory cytokines	No cytokine stress response
	High adenosine levels → Immunomodulation	Lower adenosine levels	–
Dendritic cells	–	High plasmacytoid DC (pDC:cDC ratio 3:1) in serum	High conventional DC (pDC:cDC ratio 1:3) in serum
	Low receptor levels	Low receptor levels	Normal
	Impaired vaccine response	Impaired vaccine response	Normal response
	Very low levels in tissues	Low levels in tissues	Higher levels in tissues
	Induce anti-inflammatory IL-10	Induce IL-10, IL-6, and IL-23	Induce IL1β and TNFα
	Poor antiviral response	Improved antiviral response	Intact antiviral response
	Poor induction of Foxp3(+) Treg	Poor induction of Foxp3(+) Treg	Normal induction of Foxp3(+) Treg
	Increased allergy prevalence	Increased allergy prevalence	
Neutrophils	Very low levels of L-selectin	Low levels of L-selectin <50%	Normal
	Low β2 integrin	Low β2 integrin	Normal
	Unable to diapedese	Poor diapedesis	Normal
	Diminished opsonization	Improved opsonization	Normal
	Impaired respiratory burst	Intact respiratory burst	Normal
	Very low levels of bactericidal molecules in neutrophilic granules	Low levels of bactericidal molecules in neutrophilic granules	Normal
	Poor NET formation	Poor NET formation	Normal
	No reserve in the setting of infection	No reserve in the setting of infection	Normal
MDSC	Low	High	Low
NK cells	Normal to slightly higher number	Normal to slightly higher number	Normal
	Deficient in IFNγ and TNFα production	Deficient in IFNγ and TNFα production	Normal
	About 50% do not express CD56; reduced cytotoxic function	About 50% do not express CD56; reduced cytotoxic function	Normal
	Unknown	50% ADCC compared to adults	Normal
T cells	50% lymphopenia compared to full term	Higher counts	Normal
	Low CD8 count, but CD8 cytolytic activity intact	Normal counts; Intact CD8 activity	Normal
	Impaired Th1 differentiation, favor Th2 and Th17	Impaired Th1 differentiation, favor Th2	Th1 differentiation intact
	Normal Treg levels	Normal Treg levels	Normal Treg levels
	Impaired homing of T cells to target tissues	–	Normal homing capacity
Serum Maternal IgG	10–50% of maternal levels	20–30% above maternal levels	–
	Low opsonic activity	Low opsonic activity	Normal
sIgA	Low in Serum	Higher in serum	–
	High in Mother's breastmilk	Lower in mothers breastmilk	–

Because infants with immature host innate and adaptive immune systems also have abnormal patterns of colonizing gut bacteria, there is disruption of bacterial homeostasis referred to as dysbiosis, which causes gut bacteria over-reactivity that may lead to further inflammation. The resulting high proinflammatory and pro-oxidant stress inevitably leads to irreversible damage to vital organs, including brain and intestine that often results in neurodevelopment impairment (143). Systemic inflammation during the first weeks of life is predictive of neonatal cerebral white matter injury (144), microcephaly (145), and cognitive impairment at 2 years of age (146). We have recently shown that NEC-induced brain injury, which is more severe than the brain injury that occurs in age-matched premature infants who do not develop NEC, and is characterized by significant white matter injury leading to cognitive impairment, develops as a result of cytokine release from the injured intestinal epithelium, which causes microglial activation, and the release of ROS (147). Accordingly, strategies which target the microglia and dampen the ROS response were shown in pre-clinical models to protect against the development of histologic NEC-induced brain injury, and importantly to prevent the development of cognitive impairment even in the setting of severe NEC (147).

The link between the gut and lung microbiome's development is an area of active study. Gut and lung microbiota participate in a complex interaction that shapes the host immune system, evidenced by the bidirectional association of gut dysbiosis with lung disease. For instance, infants with early life asthma have been found to have increased levels of *Clostridia* and reduced *Bifidobacteria* in the gut (148).

## Summary

The response of the compromised neonate to potential infection reflects a pattern of unique features of the premature host, that stem in part from a variety of under-developed innate and adaptive immune responses. Such responses leave the premature neonate vulnerable to significant infection, while also playing an important role in the pathogenesis of diseases that are unique to this population, including necrotizing enterocolitis, as well as the sequelae of lung and brain injury. A greater understanding of the genetic, cellular, hormonal and metabolic regulation of the immune pathways of the newborn is likely to yield novel insights into how this population responds to infection and develops disease, and will hopefully unlock new avenues for prophylaxis and therapy of newborn septic disorders.

## Author Contributions

All authors conceived, wrote, and edited the manuscript.

## Conflict of Interest

The authors declare that the research was conducted in the absence of any commercial or financial relationships that could be construed as a potential conflict of interest.
